# Electrochemically induced surface reconstruction of Ni‐Co oxide nanosheet arrays for hybrid supercapacitors

**DOI:** 10.1002/EXP.20210178

**Published:** 2021-12-16

**Authors:** Teng Wang, You Wang, Jiaqi Lei, Kai‐Jie Chen, Hongxia Wang

**Affiliations:** ^1^ Key Laboratory of Special Functional and Smart Polymer Materials of Ministry of Industry and Information Technology, Xi'an Key Laboratory of Functional Organic Porous Materials, Department of Chemistry, School of Chemistry and Chemical Engineering Northwestern Polytechnical University Xi’ an P. R. China; ^2^ School of Chemistry and Physics Faculty of Science Queensland University of Technology Brisbane Australia

**Keywords:** electrochemical activation, high energy density, hybrid supercapacitor, nanosheet array, transition metal oxides

## Abstract

Transition metal oxides (TMOs) are promising materials for supercapacitors (SCs) because of their high theoretical capacity. However, their finite active sites and poor electrical conductivity lead to reluctant electrochemical performance. Herein, we report a facile electrochemical activation (ECA) method to boost the electrochemical activity of Ni‐Co oxide nanosheet arrays (NiCoO NSA) for SCs. Specifically, honeycomb‐like NiCoO NSA that was made through a solvothermal method followed by air annealing was activated by simply exerting certain cyclic voltammetry scans (the activated sample is named ac‐NiCoO NSA). We have found this treatment results in dramatic surface structure change, forming numerous sub‐nanostructures (nanoparticles and nano‐leaves) on the NiCoO nanosheets. Rich antisite defects and oxygen vacancies in the NiCoO spinel phase were also created by the ECA treatment. Consequently, the ac‐NiCoO NSA delivered a maximum capacity of 206.5 mAh g^−1^ (0.5 A g^−1^), which is about three times of the NiCoO NSA without treatment. A hybrid SC based on the ac‐NiCoO NSA demonstrated excellent energy storage capacity (power density of 17.3 kW kg^−1^ and energy density of 45.4 Wh kg^−1^) and outstanding cyclability (>20,000 cycles, 77.4% retention rate). Our method provides a simple strategy for fabricating high performance TMOs for electrical energy storage devices like SCs.

## Graphical abstract

NiCoO nanosheet array (NSA) was converted into hierarchical nanoarrays (ac‐NiCoO NSA) through a unique electrochemical activation method. The hierarchical nanostructure, rich oxygen vacancies, antisite defects, and high surface area of ac‐NiCoO NSA led to significant electrochemical performance enhancement for supercapacitors.

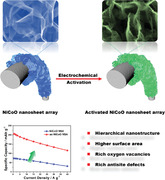

## 1 INTRODUCTION

Supercapacitors (SCs, also called electrochemical capacitors) are important energy storage devices that are featured with high safety, extraordinary long‐term cycling life, and outstanding power density. Current commercial SCs based on carbon materials deliver limited energy (<10 Wh kg^−1^) because of the electrical double‐layered capacitor‐based charge storage mechanism.^[^ ^1^ ^]^ To tackle the above bottleneck, hybrid SCs (HSCs) composed of a capacitive anode and a battery‐type cathode have attracted great attention thanks to the considerably high energy density, decent power density, and long cyclability benefiting from the respective advantages of each electrode including the Faraday charge transfer of the battery‐type cathode and the high power density of the carbon‐based anode in the charge/discharge process.^[^ ^2^ ^]^

The key for fabricating high performance HSCs is to design suitable battery‐type electrode materials with robust microstructure required for long cycling performance and high specific capacity. Nickel and cobalt oxides are considered ideal candidates for this purpose owing to their advantages of easy fabrication, low cost, and high theoretical specific capacity.^[^ ^3^ ^]^ The study of NiO and CoO*_x_* for SCs can be traced back to the 1990s when only a very limited specific capacitance (<300 F g^−1^) was demonstrated.^[^ ^4^ ^]^ The main issues with Ni/Co oxides for SCs are their poor electrical conductivity, sluggish Faradaic reaction kinetics, and large volume change during charge transfer process, resulting in low specific capacity, poor rate capability, and short charge/discharge cycle life. Therefore, it is of high importance to develop effective modification methods to alleviate those issues. A variety of methods such as hierarchical nanostructure engineering, crystal defect tuning, and composition and interface optimization have been explored.^[^ ^2a^ ^]^ Among them, the design and synthesis of array structures grown directly on conductive substrates have exhibited the effectiveness of delivering high electrical conductivity, fast electrolyte diffusion rate, and robust microstructure which can tolerate the significant volume change during the charge transfer process compared to powder‐sample‐based electrodes.^[^ ^5^ ^]^ For example, Alshareef et al. synthesized mesoporous Co_3_O_4_ nanowire arrays on carbon paper,^[^ ^6^ ^]^ which showed a high capacitance of 1525 F g^−1^ at 1.0 A g^−1^. Comparably, binary or multiple transition metal oxides (TMOs) such as NiCo_2_O_4_, ZnCoO, MnCo_2_O_4_, ZnNiCoO, and Zn‐Ni‐Al‐CoO arrays,^[^ ^7^ ^]^ can deliver higher electrochemical performance than their monometallic oxides owing to the synergistic effects of different transition metal elements. Nevertheless, further improvements in cycling stability, electrical conductivity, and specific capacity for Ni/Co oxides are still required for practical applications.

Recently, electrochemistry‐based methods have been used to enhance the capacitive properties of transition metal compounds through triggering in situ phase transformation and morphological structural change.^[^ ^8^ ^]^ For example, Qiu's group recently revealed cyclic voltammetry (CV) scanning could induce the structural transformation of NiCo carbonate hydroxide (CH). Irreversible crystal phase transformation of the pristine material into NiCo layered double hydroxide (LDH) nanosheets with abundant oxygen vacancies has been observed.^[^ ^9^ ^]^ Xia's research group reported spinel Mn_3_O_4_ nanowall arrays could be transformed into Birnessite Na_0.5_MnO_2_ hierarchical nanoarrays using the electrochemical oxidation method (200 CV scans).^[^ ^10^ ^]^ The Na_0.5_MnO_2_ arrays exhibited a wide voltage window (0–1.3 V vs. Ag/AgCl) and a much enhanced specific capacitance compared to the pristine Mn_3_O_4_. Although it is a common strategy to electrochemically activate transition metal compounds by running numerous CV cycles before the electrochemical performance test in practice, this step is mainly to ensure a better contact of the active electrode material with electrolyte without dramatic microstructure change. In most cases, only mild performance enhancement is obtained with the CV scanning treatment.

Herein, we demonstrate a unique electrochemical activation (ECA) method which could effectively activate Ni‐Co oxide nanosheet arrays (NiCoO NSA) grown on carbon fiber clothes (CFCs), leading to much enhanced electrical energy storage ability. Specifically, we exerted only 20 CV scans to the as‐prepared NiCoO NSA in a wide voltage window of −1.0 to 0.65 V (vs. Hg/HgO) in pure alkaline aqueous electrolyte (2 M KOH). The pristine NSAs turned into hierarchical nanosheet arrays (named ac‐NiCoO NSA). We also noticed the CV scanning induced a drastic change of surface elemental chemistry including the introduction of oxygen vacancies and antisite defects between Ni and Co elements. These changes have resulted in increased specific surface area, well‐exposed surface‐active sites, and efficient electrolyte ion diffusion in the ac‐NiCoO NSA. Consequently, the as‐prepared ac‐NiCoO NSA exhibited significantly improved specific capacity (206.5 mAh g^−1^ at 0.5 A g^−1^), which is almost threefold of the NiCoO NSA. We further fabricated an HSC by using the ac‐NiCoO NSA as cathode and activated carbon (AC) as anode, which delivered significantly high energy density (45.4 Wh kg^−1^), power density (17.3 kW kg^−1^), and ultra‐long cycling stability with the retention rate of 77.4% after 20,000 cycles (20 A g^−1^).

## 2 RESULTS AND DISCUSSION

**FIGURE 1:**
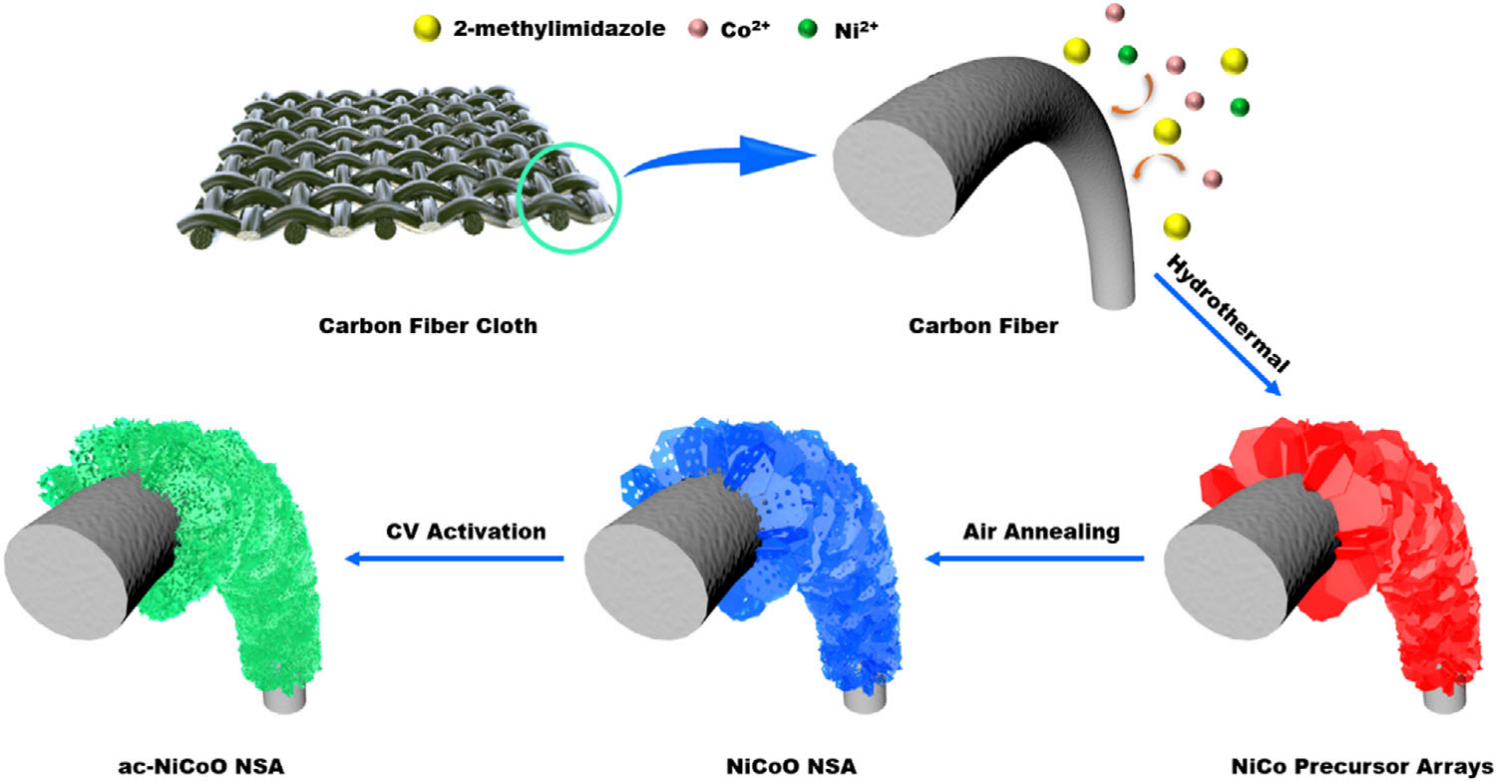
The schematic illustration of the synthetic route of ac‐NiCoO NSA grown on CFC substrate

The fabrication procedure for the ac‐NiCoO NSA material is illustrated in Figure 1. Briefly, NiCoO NSA grown on CFC substrate was firstly synthesized by air annealing of NiCo precursor arrays that were obtained through a solvothermal method. After this, the as‐prepared sample was electrochemically activated by running 20 CV scans in a 2 M KOH aqueous electrolyte. The material is named ac‐NiCoO NSA in the following. The detailed synthesis procedure is provided in the Supporting Information.

**FIGURE 2:**
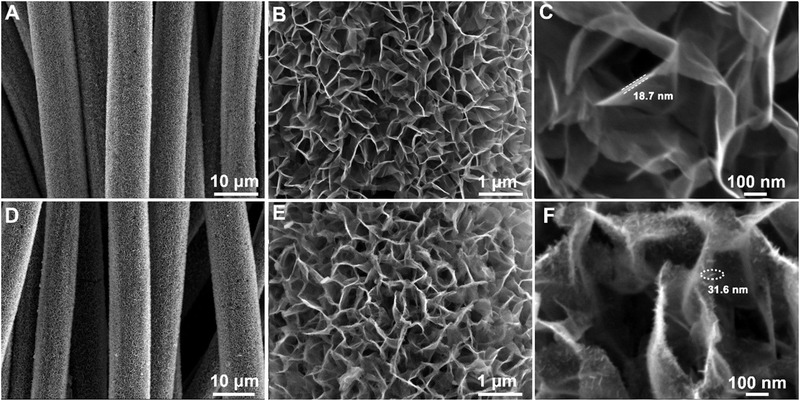
FESEM images of NiCoO NSA (A–C) and ac‐NiCoO NSA (D–F) with different magnifications

The field emission scanning electron microscope (FESEM) was employed to reveal the morphology of the products. Figure S1 shows bare carbon fibers have smooth surfaces with an average diameter of 7–8 μm. After the solvothermal reaction, a dense layer of NiCo precursor nanosheets was coated on the carbon fibers (Figure S2). After the air annealing at 350°C for fabricating NiCoO NSA, the microstructure of the NiCo precursors was well preserved (Figure 2A). The enlarged FESEM image (Figure 2B) further reveals the material consists of curved nanosheets with a honeycomb‐like porous microstructure. The pore size amongst the nanosheets is around 500 nm or below, which can reserve localized electrolytes for efficient ion diffusion. Notably, the as‐synthesized NiCoO nanosheets possess a thin thickness (<20 nm, marked in Figure 2C). For the ac‐NiCoO NSA, the main micro‐morphology of the original NiCoO NSA was retained after the ECA process (Figure 2D,E). As further revealed in Figure 2F, the nanosheets in the ac‐NiCoO NSA experienced significant surface change with the formation of numerous sub‐nanostructures (mainly nanoparticles and nano‐leaves) that are evenly distributed on the material surface. As shown in Figure S3, the sub‐nanostructures own an average lateral size of only 24.4 nm. The coarsening of the NiCoO nanosheet surface provides a large specific surface area and rich redox reaction active sites. The small size of the sub‐nanostructures benefits fast electrolyte ion diffusion amongst the porous array structure. N_2_ adsorption–desorption isotherm measurements were employed to study the surface area. As shown in Figure S4, the bare carbon cloth (CFC) exhibited negligible specific surface area while the NiCoO NSA and ac‐NiCoO NSA possess BET surface areas of 87.2 and 129.6 m^2^ g^−1^, respectively. The results prove that the ECA treatment creates more sub‐surfaces with the ac‐NiCoO NSA compared to the original NiCoO NSA sample, which is expected to benefit the rich redox reaction for efficient energy storage. The elemental composition of the ac‐NiCoO NSA measured by energy dispersive spectrometer (EDS, Figure S5) confirms that the as‐synthesized ac‐NiCoO NSA consists of Ni, Co, and O with the atomic ratio of Co/Ni = 1. The EDS signal of C comes from the CFC substrate and the detected Al is originated from the aluminum sample stage. Moreover, the elemental mapping confirms the even dispersion of Ni, Co, and O elements in the ac‐NiCoO NSA (Figure S6). The EDS and elemental mapping of NiCoO NSA gave similar results in terms of the content and elemental dispersion of Ni and Co in the ac‐NiCoO NSA (Figures S7 and S8). To get a more accurate atomic ratio of Ni and Co in both oxide materials, we carried out inductively coupled plasma‐optical emission spectrometry (ICP‐OES) measurements. The results show that NiCoO NSA has an atomic ratio of Ni/Co of 1.27 which is slightly higher than that of the ac‐NiCoO NSA (atomic ratio of Ni/Co = 1.17). This indicates that the ECA process probably caused partial dissolution and rearrangement of transition metal ions in the material, in particular on the material surface. Based on the ICP‐OES measurement, we determined the accurate chemical formula for both materials which is Ni_1.68_Co_1.32_O_4_ for NiCoO NSA and Ni_1.62_Co_1.38_O_4_ for ac‐NiCoO NSA, respectively.

**FIGURE 3:**
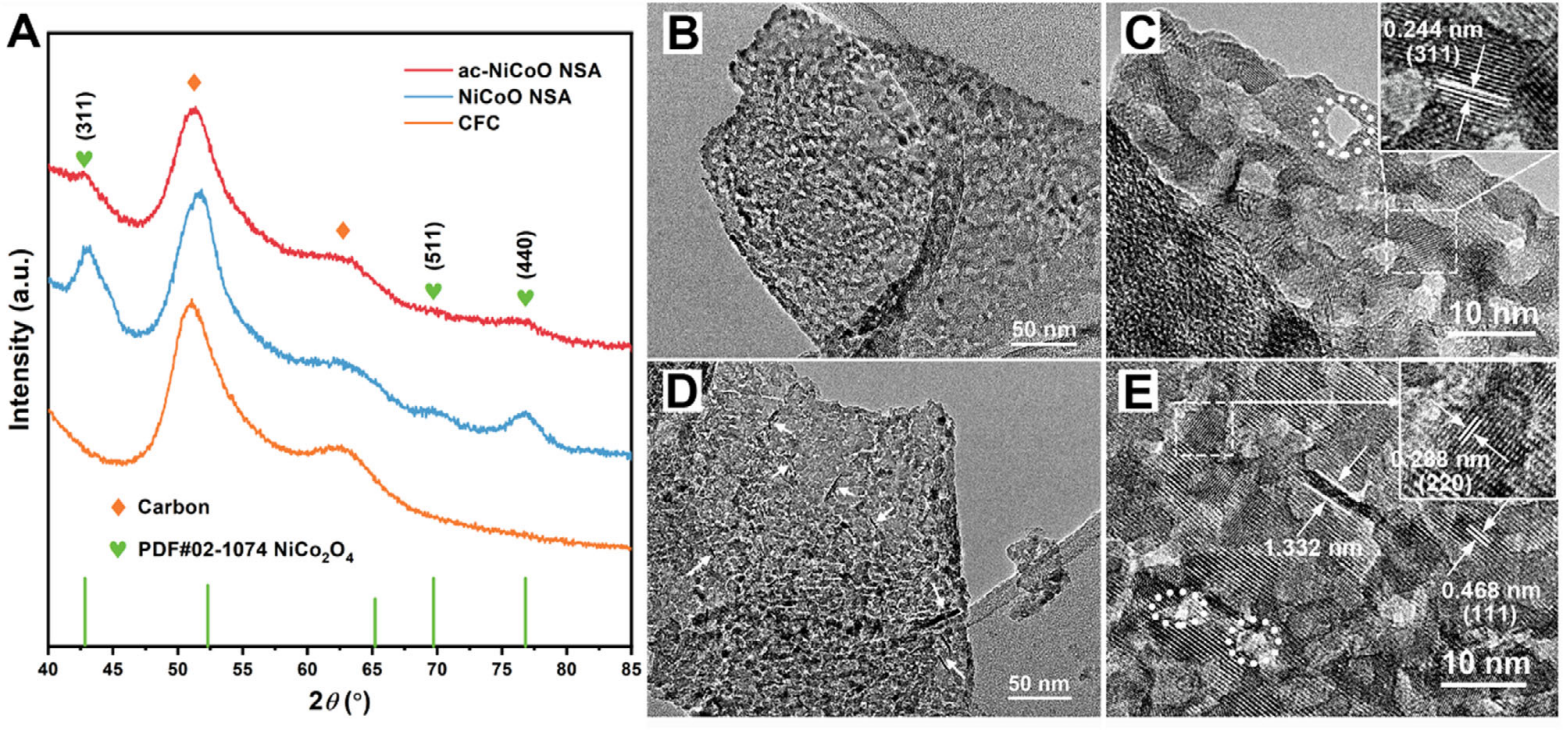
(A) PXRD patterns of bare CFC substrate, NiCoO NSA, and ac‐NiCoO NSA. TEM images of NiCoO NSA (B, C) and ac‐NiCoO NSA (D, E). The insets are the corresponding enlarged lattice fringe information

Powder X‐ray diffraction (PXRD) was employed to obtain information on the crystal structure of the as‐obtained samples. As seen in Figure 3A, the PXRD patterns of NiCoO NSA and ac‐NiCoO NSA are nearly identical except the reduced peak intensity of ac‐NiCoO NSA, suggesting the ECA process led to an unchanged crystal structure of the material. This phenomenon differs from the previous reports in which the ECA induced material phase change.^[^ ^8^ ^]^ It is also found that the three characteristic diffraction peaks at 42.8°, 69.7°, and 76.8° can be indexed to the (311), (511), and (440) facets of spinel phase NiCo_2_O_4_ (PDF#02‐1074). We further investigated the structure and morphology of the material using a transmission electron microscope (TEM). Specifically, the TEM image (Figure 3B) shows a clear porous nanosheet morphology of the raw NiCoO while small nanopores can be observed in the high resolution (HR) TEM image (highlighted by the dot line circles in Figure 3C). Obvious lattice fringes with lattice space (inset of Figure 3C) of 0.244 nm were detected, which is attributed to the (311) facet of the spinel phase (PDF#02‐1074). This is consistent with the PXRD results. After the ECA step, the ac‐NiCoO NSA maintained a similar porous nanostructure except that numerous sub‐nanostructures including nanoparticles and nano‐leaves were formed on the material surface (Figure 3D). It is seen in Figure 3E that the nanopores on the nanosheets turned much smaller (highlighted by the white circles) after the ECA step. The newly formed nano‐leaves (Figure 3E) contain only three atomic layers with a thickness of a. 1.33 nm. Furthermore, the atomic force microscope (AFM) image and the corresponding thickness disperse curve of ac‐NiCoO NSA reveal the main nanosheets also have an ultrathin thickness of 1.30 nm (Figure S9). The ultrathin nanostructures mean a very high exposure of the active metal sites for redox reactions, which favors the energy storage of materials. The measured lattice fringes exhibit space values of 0.288 nm (inset of Figure 3E) and 0.468 nm (Figure 3E), respectively, which are indexed to the (220) and (111) facets of the spinel phase.

**FIGURE 4:**
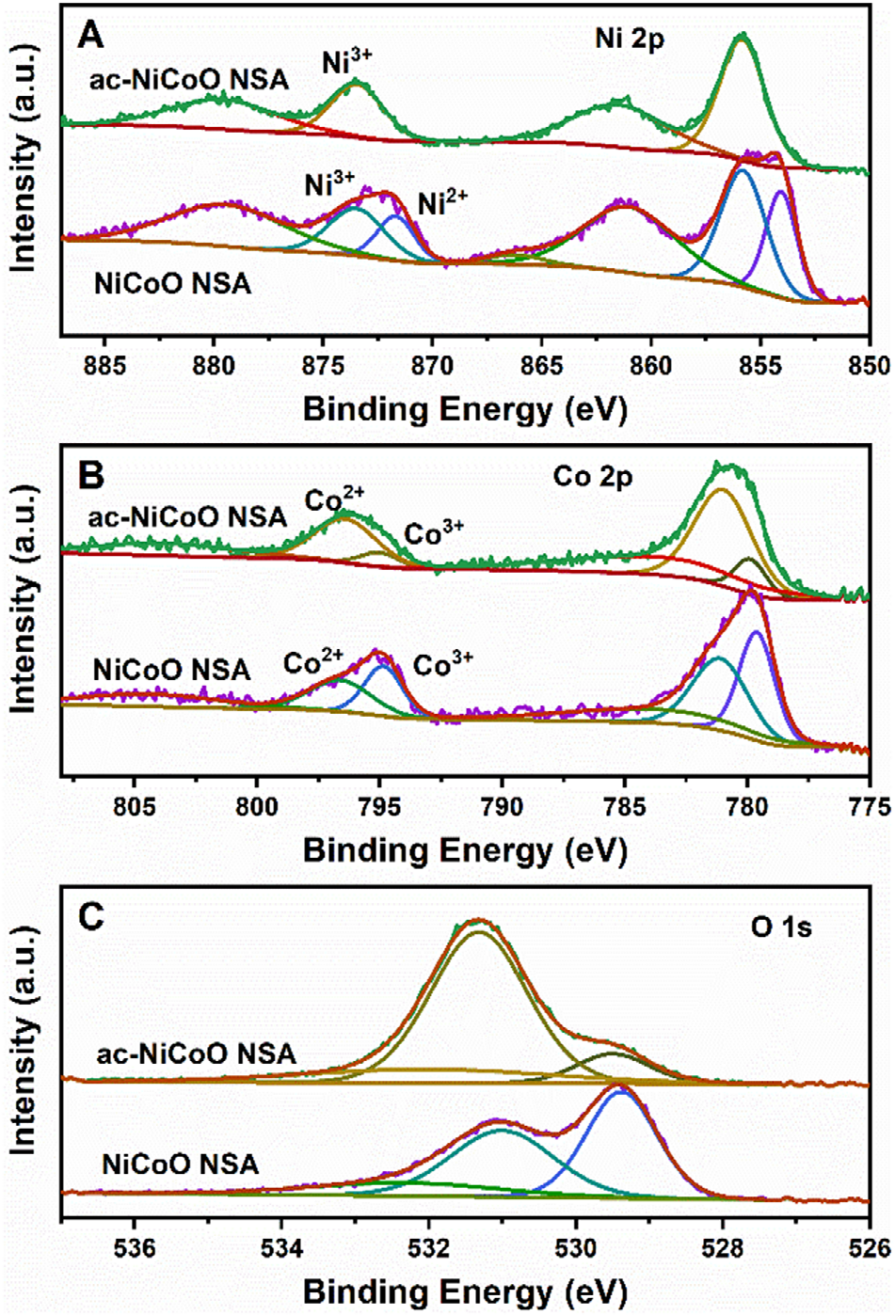
Deconvoluted XPS spectra of Ni 2p (A), Co 2p (B), and O 1s (C) in NiCoO NSA and ac‐NiCoO NSA

The surface chemical composition of the material also plays a critical role in the electrochemical properties. The full survey spectra of X‐ray photoelectron spectroscopy (XPS) of both NiCoO NSA and ac‐NiCoO NSA (Figure S10) confirm the elemental composition of Co, Ni, and O, which is in accordance with the EDS results above. Deconvolution of the XPS spectra (Figure 4) of Co 2p, Ni 2p, and O 1s reveals that the surface elements of the sample experienced significant change after the ECA process. As shown in Figure 4A, the main XPS spectrum of Ni 2p_1/2_ spin‐orbit in pristine NiCoO NSA can be fitted with two peaks at 871.75 and 873.50 eV, respectively, which correspond to the characteristic peaks of Ni^3+^ and Ni^2+^, respectively.^[^ ^11^ ^]^ By contrast, the Ni 2p_1/2_ XPS spectrum of the ac‐NiCoO NSA shows a drastically positive shift and only one characteristic peak at 873.50 eV, which is attributed to Ni^3+^ occupied in the octahedral site of spinel phase.^[^ ^12^ ^]^ This indicates all surface Ni elements have been oxidized to 3+ valence state after the ECA process. The XPS spectra of Co 2p in both samples suggest a mixed‐valence state of 2+/3+ in Co (Figure 4B). In the Co 2p_1/2_ spectra, the peak at the binding energy of 796.45–796.70 eV is indexed to the Co^2+^ and the one at 794.76–795.01 eV is attributed to the Co^3+^.^[^ ^11^ ^]^ Based on the XPS fitting results, the atomic ratios of Co^2+^/Co^3+^ in NiCoO NSA and ac‐NiCoO NSA were calculated to be 1.0 and 5.0, respectively. Clearly, most cobalt cations in the original NiCoO NSA have been electrochemically reduced to 2+ valence state after the activation process. As for the XPS spectra of O 1s (Figure 4C), three fitting peaks at 532.14–532.41, 531.03–531.30, and 529.38–529.47 eV can be assigned to the surface‐adsorbed water molecule, formation of O─Ni^3+^ bond in the octahedral site and oxygen vacancies, and the lattice O in spinel oxide, respectively.^[^ ^12^, ^13^ ^]^ These results further confirm the increase of Ni^3+^ and Co^2+^ in the ac‐NiCoO NSA sample compared to pristine NiCoO NSA, suggesting the formation of enriched antisite defects in the spinel phase since the valence state change of the metal ions means the shift of their occupation sites in the crystal. The change of valence state of Ni and Co ions implies a charge transfer between the two metal ions (Ni^2+^ + Co^3+^ → Ni^3+^ + Co^2+^) during the ECA process. Theoretically, the electrochemical potential of Co^3+^/Co^2+^ (1.81 V vs. NHE) is much higher than Ni^3+^/Ni^2+^ (0.49 V), which makes the oxidation of Ni^2+^ by Co^3+^ possible under appropriate reaction conditions like the ECA process. It has been reported that Ni^3+^ and rich oxygen deficiencies are highly active for generating redox reactions for electric energy storage while cobalt oxide can provide a robust matrix for efficient charge transportation.^[^ ^12^ ^]^ Therefore we anticipate that the drastically irreversible change of surface elemental chemistry of NiCoO NSA may favor the electrochemical performance of the materials in SCs.

**FIGURE 5:**
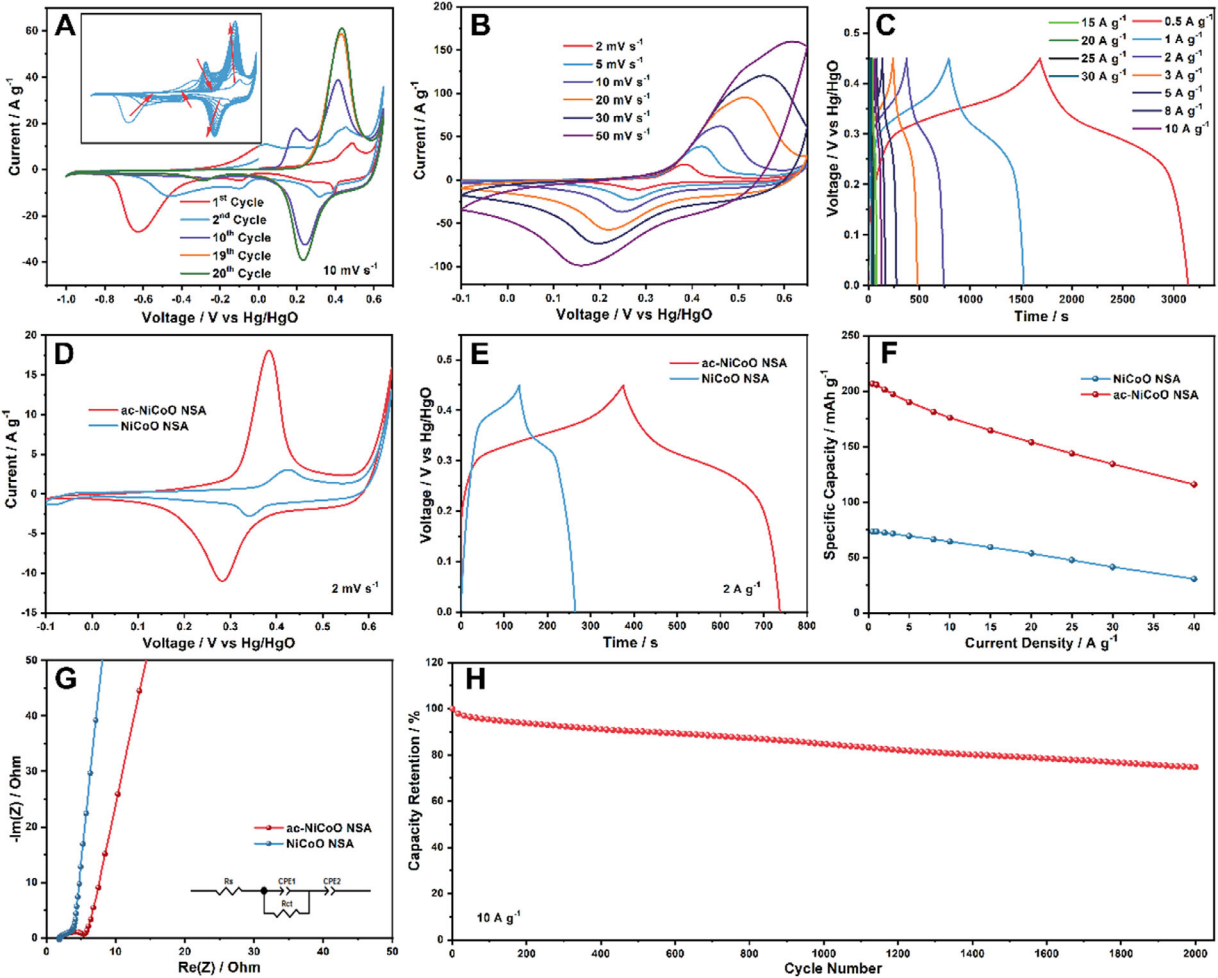
(A) The CV plots at 1st, 2nd, 10th, 19th, and 20th CV cycles of NiCoO NSA at 10 mV s^−1^ during the ECA process. The inset shows the CV plots of all 20 CV scans with the red arrows showing the redox peaks evolution direction. (B) CV and (C) galvanostatic charge/discharge (GCD) curves of the ac‐NiCoO NSA. The comparison of CV (D), GCD (E), specific capacity (F), and Nyquist impedance (G) curves of the NiCoO NSA and ac‐NiCoO NSA. (H) The cycling results of ac‐NiCoO NSA under 10 A g^−1^

To understand this phenomenon, we monitored the CV plots of the pristine NiCoO NSA during the ECA process in a three‐electrode setup with 2 M KOH aqueous electrolyte (more details are shown in the Supporting Information). The ECA of NiCoO NSA was proceeded by running 20 CV cycles in the voltage range of −1.0 to 0.65 V vs. Hg/HgO. As indicated by Figure 5A and the red arrows in the inset, the NiCoO NSA experiences irreversible redox reactions along with the CV scanning. It is noticed the irreversible redox peaks in the negative voltage range disappeared completely at the 20th CV scan while the intensity of a pair of reversible redox peaks at 0.23 and 0.43 V increased continuously. It suggests the complete transformation of pristine NiCoO NSA to ac‐NiCoO NSA at the 20th CV cycle. Since all irreversible redox reaction peaks disappeared and the reversible redox peaks at the positive voltage window tend to reach the maximum point, 20 CV cycles were chosen as the optimized activation condition. Based on the XPS results, the irreversible reactions should be attributed to the charge transfer between Ni and Co elements. To confirm this, we also conducted the conventional ECA method for activating NiCoO NSA. As shown in Figure S11, the CV scans in the positive range (0–0.65 V, 20 cycles) only resulted in a medium increase of the reversible redox peak current without any irreversible redox peaks observed, indicating a medium increase of the electrochemical property. In comparison, NiO and Co_3_O_4_ NSA on CFC were also activated at the same voltage window (−1.0 to 0.65 V, Figure S12). We did not observe significant changes in the CV plots of NiO and Co_3_O_4_, proving that only binary NiCoO NSA can be electrochemically activated in this unique way, which is probably ascribed to the synergistic effects of Ni and Co elements.

The energy storage properties of all samples were further evaluated. As shown in Figure 5B, all the CV curves of ac‐NiCoO NSA depict a distinct pair of redox peaks with a large potential polarization, implying the battery‐type charge storage mechanism.^[^ ^14^ ^]^ The CV plots of NiCoO NSA also showed obvious redox peaks (Figure 5D). The possible redox reactions of the NiCoO NSA and ac‐NiCoO NSA that occurred during the CV scanning are shown in Equations (1) and (2)^[^ ^15^ ^]^:

(1) $$\begin{array}{ccc} & & Ni_{x}Co_{3-x}O_{4}\left(x=1.0-2.0\right)+OH^{-}+H_{2}O↔x\mathrm{NiOOH}\\ & & \;+\left(3-x\right)\mathrm{CoOOH}+2e^{-} \end{array}$$

(2) $$\mathrm{CoOOH}+{\mathrm{OH}}^{-}↔\mathrm{Co}O_{2}+H_{2}O+e^{-}$$

Ni*_x_* Co*_3− x_* O_4_ (*x* = 1.0 – 2.0) represents the composition of both oxide materials (NiCoO NSA and ac‐NiCoO NSA). Figure 5B also shows the redox peaks only mildly shift with the increase of sweep rates, which still exist even at 50 mV s^−1^, indicating the highly reversible charge process and high rate capability. The galvanostatic charge/discharge (GCD) curves (Figure 5C) of the as‐obtained ac‐NiCoO NSA (from 0.5 to 30 A g^−1^) show obvious voltage plateau and symmetric shape, further confirming its outstanding charge storage capacity and rate performance. The voltage plateau indicates the typical battery‐type charge transfer mechanism, which agrees with the CV plots. The possible redox reaction should be mainly attributed to the reaction of Co^2+/3+/4+^ (as shown in Equations 1 and 2). We have employed ex situ XPS to further study the surface chemistry evolution of Ni and Co metal ions in the ac‐NiCoO NSA at different charge/discharge states (Figure S13). More specifically, the XPS spectra of Ni 2p show nearly constant characteristic peak locations (Figure S13B) with the peak of Ni 2p_1/2_ positioned at 873.23 eV, indicating the nearly unchanged valence state of Ni^3+^. On the contrary, the Co 2p spin‐orbit exhibits varied XPS spectra along with the change of the charge/discharge states (Figure S13C). It is seen that the characteristic peak of Co 2p_1/2_ negatively shifted from 795.77 to 794.90 eV when the ac‐NiCoO NSA was charged from 0.10 to 0.45 V, indicating the oxidation of Co. When the material was discharged back to 0.10 V, the peak position for Co 2p_1/2_ positively shifted to 795.27 eV, confirming the reduction reaction of Co. In summary, the ex situ XPS results suggest that the charge storage of ac‐NiCoO NSA is mainly ascribed to the redox reactions of Co ions while Ni remained a relatively stable charge state of 3+, which matches with our proposed reactions as shown in the equations above. It is worth pointing out that the redox reaction of Co was not fully reversible according to the ex situ XPS results.

As observed in Figures 5D,E, the ac‐NiCoO NSA possesses a much larger integrated CV area (at 2 mV s^−1^) and nearly three times longer GCD time (at 2 A g^−1^) than the NiCoO NSA (Figure S14), confirming its superior electrical storage capacity. The specific capacity of the material was calculated based on the GCD plots. As depicted in Figure 5F, the ac‐NiCoO NSA delivered a specific capacity of 206.5 mAh g^−1^ at 0.5 A g^−1^, which is nearly three times of the non‐activated NiCoO NSA (only 73.3 mAh g^−1^). Even at a high discharge current density of 40 A g^−1^, the material still maintained a capacity of 115.9 mAh g^−1^ (56.1% retention rate), which is superior to the NiCoO NSA (41.8%). To further investigate the reason for the excellent electrochemical performance of ac‐NiCoO NSA, the electrochemical impedance spectroscopy (EIS) data of the materials were collected and the corresponding Nyquist plots are shown in Figure 5G. The intersection point with the X‐axis and the diameter of the first semicircle of the Nyquist plot represent the series resistance (*R*_s_) and charge resistance (*R*_ct_) of the material, respectively.^[^ ^14^ ^]^ Obviously, the ac‐NiCoO NSA has a smaller *R*_s_ = 1.79 Ω and *R*_ct_ = 4.57 Ω than the pristine NiCoO NSA (*R*_s _= 1.81 Ω;*R*_ct _= 8.84 Ω). The decreased *R*_s_ suggests a higher electrical conductivity and the much smaller *R*_ct_ indicates a faster redox reaction kinetics, which is desirable to achieve enhanced specific capacity and rate capability. The cyclability of ac‐NiCoO NSA was measured by running GCD cycles at 10 A g^−1^. Figure 5H and Figure S15 reveal a capacity retention rate of 74.66% (112.94 mAh g^−1^) after 2000 cycles. The retention rate of ac‐NiCoO NSA is lower than the inactivated NiCoO NSA (84.60%, from 64.77 to 54.78 mAh g^−1^, Figure S16). To understand the underlying mechanism for reduced capacity, we measured the morphology and structure of the ac‐NiCoO NSA after the cycling test. The FESEM images (Figure S17) show mild destruction and surface coarsening of the hierarchical nanostructure of ac‐NiCoO NSA after the cycling test. The PXRD pattern of ac‐NiCoO NSA after the cycling test (Figure S18) shows a decrease of the characteristic peaks of spinel crystal without the formation of new peaks. Based on the above results, we ascribed the mild cycling performance decay to the irreversible redox reactions and partial destruction of the crystal structure and microstructures of the material. Nevertheless, the absolute capacity value of ac‐NiCoO NSA is still much higher than that of the pristine NiCoO NSA after the cycling test. This indicates the structural reconstruction by our ECA method mainly contributes to enhancing the electrochemical activity of the material. We also measured the electrode materials at a large current density. It is found that the capacity retention rate of ac‐NiCoO NSA was 89.3% under the voltage window of 0–0.5 V at 50 A g^−1^ (Figure S19). Clearly, the less utilization of bulk of the active material during large current cycling test favors the structural stability of the ac‐NiCoO NSA.

**FIGURE 6:**
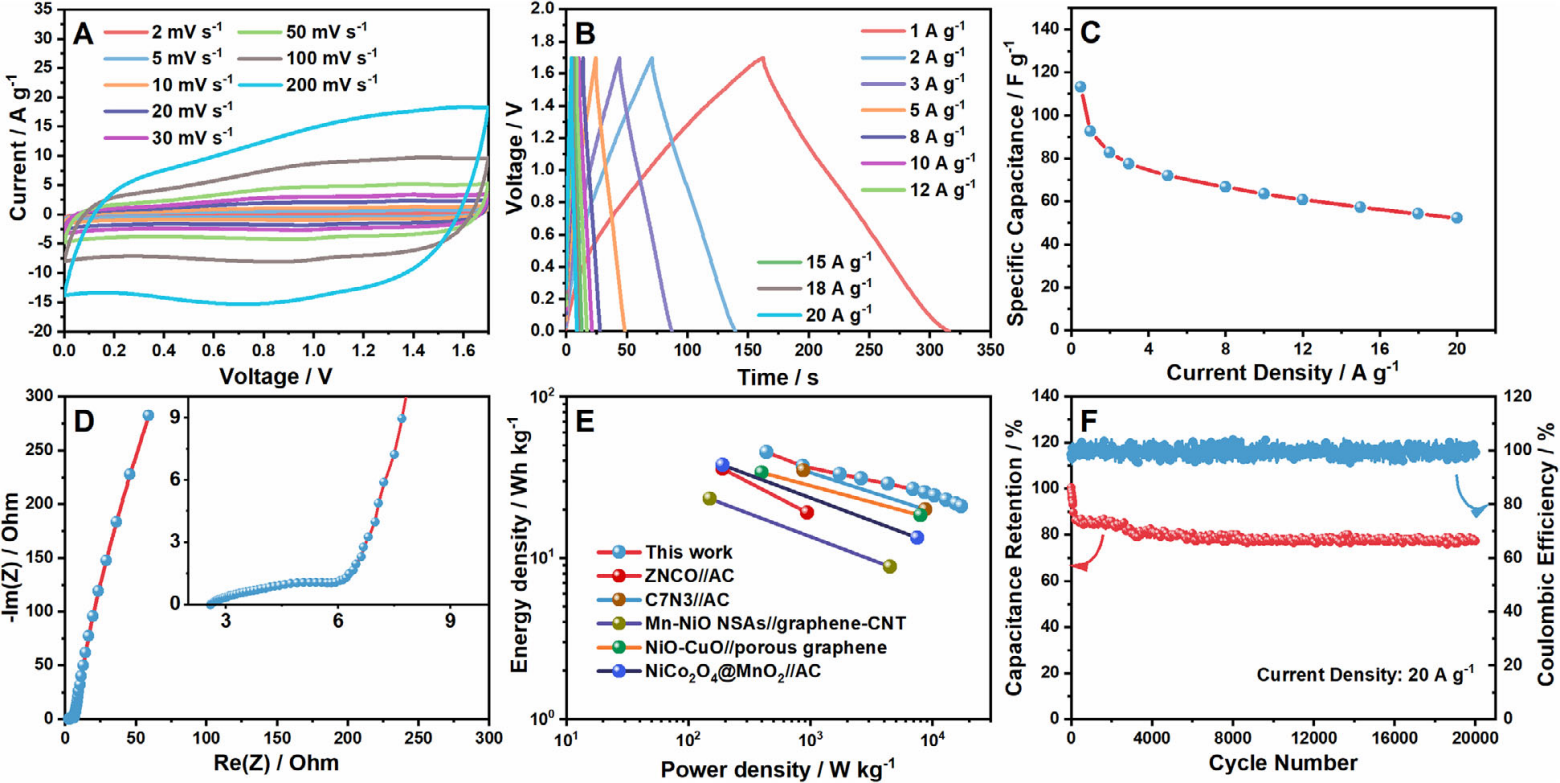
Capacitive properties of the ac‐NiCoO NSA//AC HSC: (A) CV curves, (B) GCD plots, (C) specific capacitance results, (D) Nyquist impedance plots, (E) Ragone plots, and (F) GCD cycling tests under 20 A g^−1^

The three‐electrode tests suggest the ac‐NiCoO NSA can serve as ideal electrodes for electrical energy storage devices. To confirm it, we fabricated a hybrid supercapacitor (HSC) by using the ac‐NiCoO NSA and commercially available activated carbon (AC) on Ni foam substrate as cathode and anode, respectively. Our previous report has shown the AC electrode owned a high specific capacitance of 187.7 F g^−1^ at 3 A g^−1^ and considerable rate performance (93.6% retention rate at 30 A g^−1^).^[^ ^11a^ ^]^ Regarding the assembled ac‐NiCoO NSA//AC HSC, the CV curves (Figure 6A) show that the HSC could operate safely within a voltage range from 0 to 1.7 V and no obvious electrolyte decomposition was detected. Moreover, the corresponding CV curve still displays a quasi‐rectangular shape even at a high sweep rate (200 mV s^−1^). The GCD curves (Figure 6B) under different current densities possess similar quasi‐triangular shapes and negligible IR drop. The CV and GCD results indicate the electrical charge storage of the HSC is dominated by the capacitive charge storage mechanism. According to the GCD results, the HSC delivered a specific capacitance of 113.1 F g^−1^ at 0.5 A g^−1^ (Figure 6C), which reduced to 53.1 F g^−1^ at 20 A g^−1^ (46.9% retention rate), implying a high electrical storage ability and considerable rate performance. The Nyquist plot of the device is shown in Figure 6D. Fitting the Nyquist with an equivalent circuit reveals the ac‐NiCoO NSA//AC HSC has a small *R*_s_ of 2.47 Ω and *R*_ct_ of 6.21 Ω. The small values indicate a low device internal resistance and fast charge transfer process. The Ragone diagram shows the HSC delivers the energy density of 45.4 and 20.9 Wh kg^−1^ at the power density of 436.4 W kg^−1^ and 17.3 kW kg^−1^ (Figure 6E), respectively. In the long‐term cyclability test, over 77.4% of the capacitance retention rate was achieved with the HSC after 20,000 GCD cycles under 20 A g^−1^ (Figure 6F), which is outstanding for HSCs using battery‐type electrode materials. Notably, the cell experienced quick capacitance reduction (around 15.1%) during the first 300 GCD cycles. The initial fast capacity decay should be caused by the peeling of loose particles of both electrode active materials. After this, the device became very stable. There is merely 0.3‰ capacitance loss per cycle in the later cycling test, confirming the ultra‐long cyclability of the ac‐NiCoO NSA//AC HSC. Moreover, the almost 100% Coulombic efficiency proves its considerably high transfer efficiency. As depicted in Table S1, the energy storage ability of our device is superior to many of the state‐of‐the‐art HSCs based on TMOs such as ZNCO//AC, C7N3//AC, Mn‐NiO NSAs//graphene‐CNT, NiO‐CuO//porous graphene, (MgCo_2_O_4_@PPy/NF) //AC, and NiCo_2_O_4_@MnO_2_//AC,^[^ ^16^ ^]^ suggesting the great potential of ac‐NiCoO NSA for practical applications.

We believe that there are several reasons which contribute to the enhanced electrochemical performance of ac‐NiCoO NSA: (1) formation of favorable surface sub‐nanostructures (nanoparticles and nano‐leaves) through the structural reconstruction, providing high porosity and large accessible surface area for redox reactions; (2) the binder‐free array structure endows the material a high electrical conductivity for fast charge transfer for energy storage; (3) the stable spinel crystal structure provides the material high structural stability; (4) the formation of rich electrochemically active sites for Faradaic charge storage including abundant antisite defects and oxygen vacancies.

## 3 CONCLUSIONS

In summary, we demonstrated a simple ECA method to boost the electrochemical performance of NiCoO NSA. We have found that the ECA process resulted in a significant surface reconstruction of the NiCoO NSA with desirable morphological structure and active sites for electrochemical charge transfer. In particular, the ECA process led to the formation of numerous sub‐nanostructures including nanoparticles and atomically thin nano‐leaves, and the introduction of rich antisite defects and oxygen vacancies. Owing to the unique material structure, the as‐obtained ac‐NiCoO NSA delivered a higher capacity of 206.5 mAh g^−1^ at 0.5 A g^−1^ as well as a higher rate capability (56.1% retention rate at 40 A g^−1^) than pristine NiCoO NSA. The ac‐NiCoO NSA‐based HSC device exhibited high energy density (45.4 Wh kg^−1^), power density (17.3 kW kg^−1^), and ultra‐long cyclability of 20,000 GCD cycles. The ECA can be potentially used as a universal strategy for fabricating high performance TMOs for advanced SCs.

## CONFLICT OF INTEREST

Hongxia Wang is a member of the *Exploration* editorial board. The authors declare no conflict of interest.

## Supporting information

Supporting Information
