# Supplementary material for: Electrochemically induced surface reconstruction of Ni‐Co oxide nanosheet arrays for hybrid supercapacitors
Source: Exploration (Beijing). 2021 Dec 16;1(3):20210178. doi: 10.1002/EXP.20210178 (PMC10190942; doi:10.1002/EXP.20210178)
Supplement: Supplementary file 1 — Supporting Information [file EXP2-1-20210178-s001.docx]

**Supporting Information**

**Electrochemically Forced Surface Reconstruction of Ni-Co Oxide Nanosheet Arrays for Hybrid Supercapacitors**

Teng Wang^1,2^*, You Wang^1^, Jiaqi Lei^1^, Kai-Jie Chen^1^*, and Hongxia Wang^2^*

^1^ *Key Laboratory of Special Functional and Smart Polymer Materials of Ministry of Industry and Information Technology, Xi'an Key Laboratory of Functional Organic Porous Materials, Department of Chemistry, School of Chemistry and Chemical Engineering, Northwestern Polytechnical University, Xi’ an, Shaanxi 710072, PR China. Email: wangt42@nwpu.edu.cn; ckjiscon@nwpu.edu.cn.*

^2^ *School of Chemistry and Physics, Faculty of Science,* *Queensland University of Technology, Brisbane, QLD 4001, Australia. E-mail: hx.wang@qut.edu.au.*

Experimental

- 1. Preparation of NiCoO NSA

Briefly, 40 mM 2-methylimidazole (MIM) methanol solution was added into another methanol solution containing 10 mM Ni(NO_3_)_2_·6H_2_O and 10 mM Co(NO_3_)_2_·6H_2_O under magnetic stirring. After that, the reaction solution was put into a Teflon liner stainless-steel vessel with a piece of pre-cleaned carbon fiber cloth (CFC, 1 × 4 cm^2^) for a solvothermal reaction at 140 ^o^C for 14 h. Then the product was collected and rinsed with efficient methanol and dried at 70 ^o^C in an electric air oven. Note that single nickel hydroxide and cobalt hydroxide precursor arrays grown on CFC were also synthesized by changing the metal precursor into 20 mM Ni(NO_3_)_2_·6H_2_O and 20 mM Co(NO_3_)_2_·6H_2_O, respectively. The as-prepared products were annealed at 350 ^o^C for 30 min with the ramping rate of 5 ^o^C min^-1^, resulting in the NiCoO NSA, NiO NSA, and Co_3_O_4_ NSA.

- 1. Preparation of ac-NiCoO NSA

The ECA process of the as-prepared NiCo-LDH was completed by simply running 45 cyclic voltammetry (CV) cycles from -1.0 V to 0.65 V vs Hg/HgO with the scan rate of 10 mV s^-1^ in the alkaline electrolyte (2 M KOH).

- 1. Material characterization

The characterization of the as-prepared materials has been carried out by Field emission scanning electron microscope (FESEM, Verios G4, FEI) with an energy dispersive spectrometer (EDS), powder X-ray diffraction (XRD, PANaytical) using Co Kα source, transmission electron microscope (TEM, Talos F200X, FEI), X-ray photoelectron spectroscopy (XPS, Axis Supra, Kratos) with Al (Kα =1486.6eV) X-ray as excitation source, atomic force microscope (AFM, Dimension Icon, Bruker), N_2_ gas sorption test (Micromeritics 3Flex), and Inductively coupled plasma - optical emission spectrometry (ICP-OES).

- 1. Electrochemical Measurements

The electrochemical performance of the electrodes has been measured via a three-electrode system and all the data were collected by an electrochemical workstation (VSP, BioLogic). The free-standing products including NiCoO NSA and ac-NiCoO NSA, standard Hg/HgO electrode, and a piece of Pt foil (1 × 2 cm^2^) were used for working electrode, reference electrode, and counter electrode, respectively. The cyclability of the working electrodes was measured by an automatic charge/discharge testing set (CT-4008, NEWARE).

Galvanostatic charge/discharge (GCD) tests were proceeded and used to calculate the specific capacity Q (mAh g^-1^) according to equation (1):^[1]^

$$Q=\frac{I\int_{t_{i}}^{t_{f}} Vⅆt}{1.8\times mU} (1)$$

Where *t_i_* and *t_f_* represent the initial and final values of the discharge time *t* (s). *I* (A), *m* (g), *V* (V), and *U* (V) are the discharge current, active material mass loading, operating potential, and the working potential window, respectively.

An HSC consisted of ac-NiCoO NSA based positive electrode and commercial activated carbon (AC) based negative electrode was fabricated for practical application using 2 M KOH as the electrolyte. The as-grown ac-NiCoO NSA was directly used as the positive electrode and the AC electrode was synthesized using the following procedure: the power samples of AC, carbon black, and PVDF with a mass ratio of 8: 1: 1 were thoroughly mixed and then dispersed in N-methyl pyrrolidinone (NMP) solvent for obtaining a uniform slurry. The Ni foam substrate was coated by the slurry and dried in a vacuum oven at 110 ^o^C for 12 h before being pressed under 10 MPa pressure to get the final electrode.

The specific capacitance *C* (F g^-1^) of the AC electrode and the HSC was also obtained through their corresponding GCD results based on equation (2)^[2]^:

$$C=\frac{I\Delta t}{m\Delta V} (2)$$

Where *I* (A), ∆*t* (s), *m* (g), and ∆*V* (V) mean the discharge current, discharge time, the total mass loading of active materials, and voltage window, respectively.

In order to optimize the energy storage capability, the mass ratio of both electrodes in the as-assembled HSC was well adjusted according to equation (3):

$$\frac{m_{+}}{m_{-}}=\frac{C_{-}\times\Delta V_{-}}{Q_{+}} (3)$$

Where *C_-_* (F g^-1^), ∆*V_-_* (V) and *m_-_* (g) are the specific capacitance, voltage window and mass loading of AC material. *Q_+_* (mAh g^-1^) and *m_+_* (g) stand for the specific capacity and the total mass of NiCoO NWSA active material excluding the substrate.

The energy density (*E*, Wh kg^-1^) and power density (*P*, W kg^-1^) of the device were calculated based on equations (4) and (5):^[1-2]^

$$E=\frac{I\int_{t_{i}}^{t_{f}} V_{s}ⅆt}{3.6M} (4)$$

$$P=\frac{3600\times E}{\Delta t} (5)$$

Where *I* (A), *V_s_* (V), and *∆t* (s) mean the discharge current, operating voltage, and discharge time of the as-prepared HSC. *M* (g) stands for the total mass loading of both electrodes and *t_i_* and *t_f_* represent the initial and final values of the discharge time during discharge process.


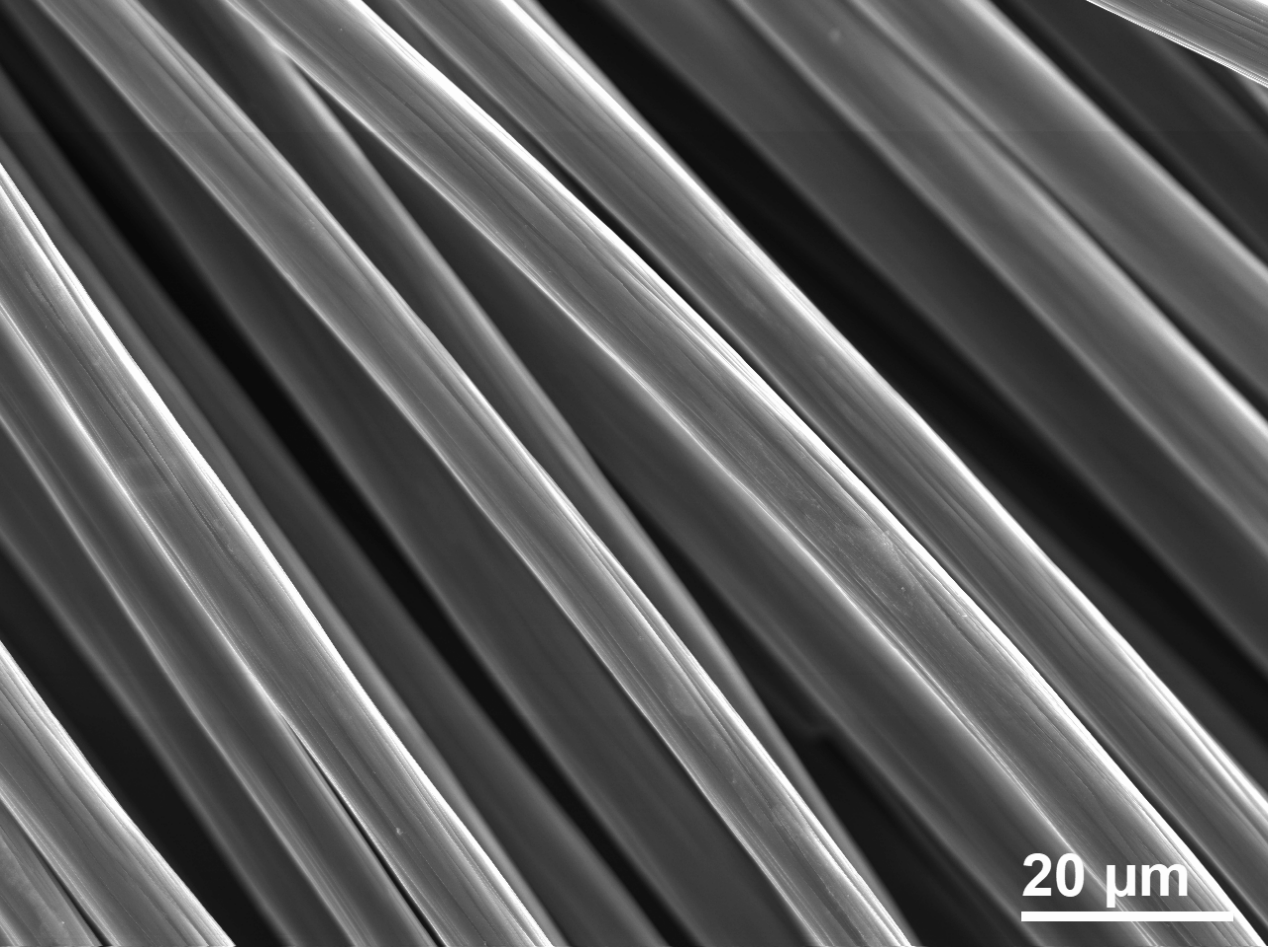


**Figure S1.** SEM image of bare CFC substrate.

**
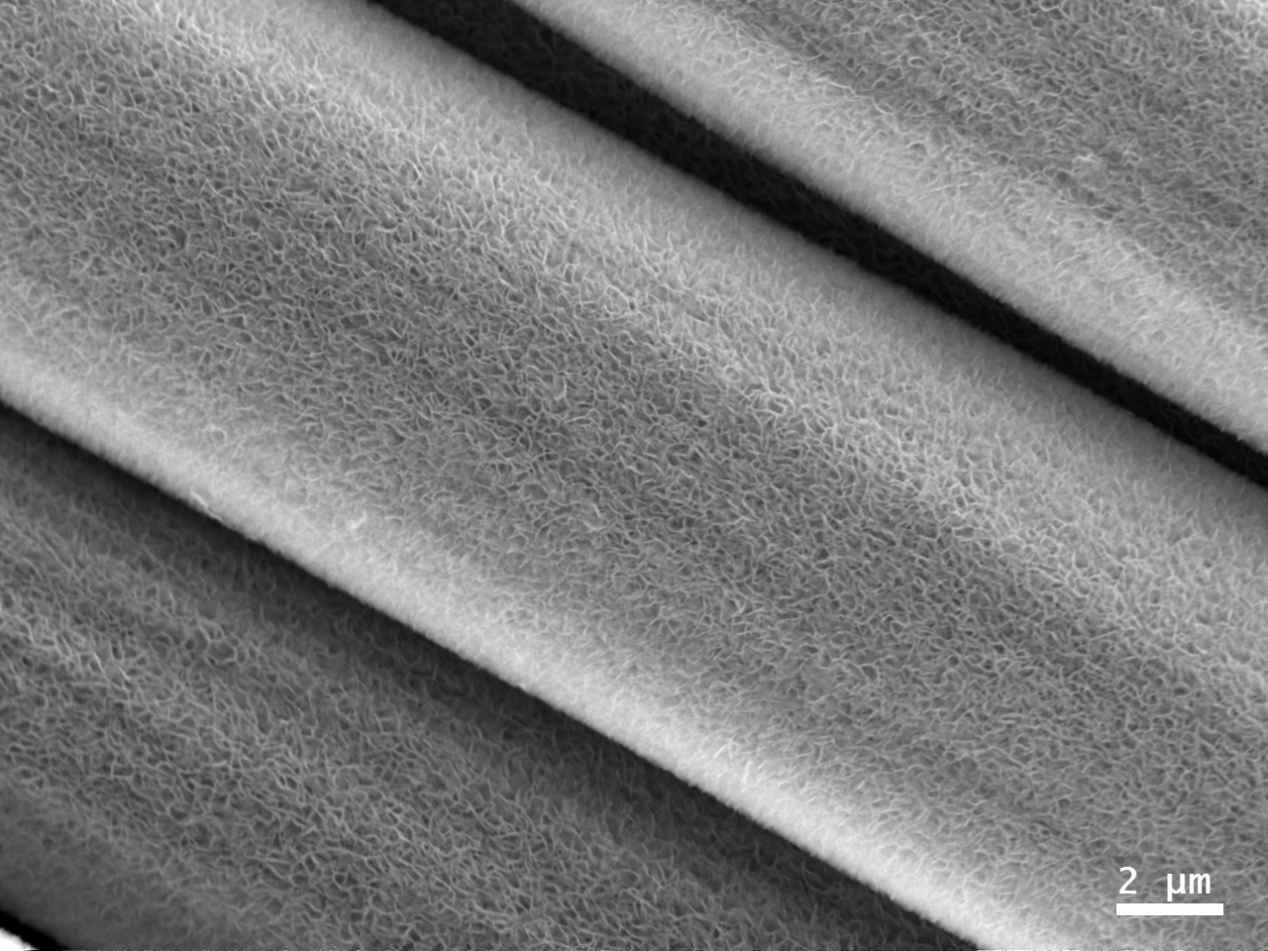
**

**Figure S2.** SEM images of NiCo precursor nanosheet arrays.

**
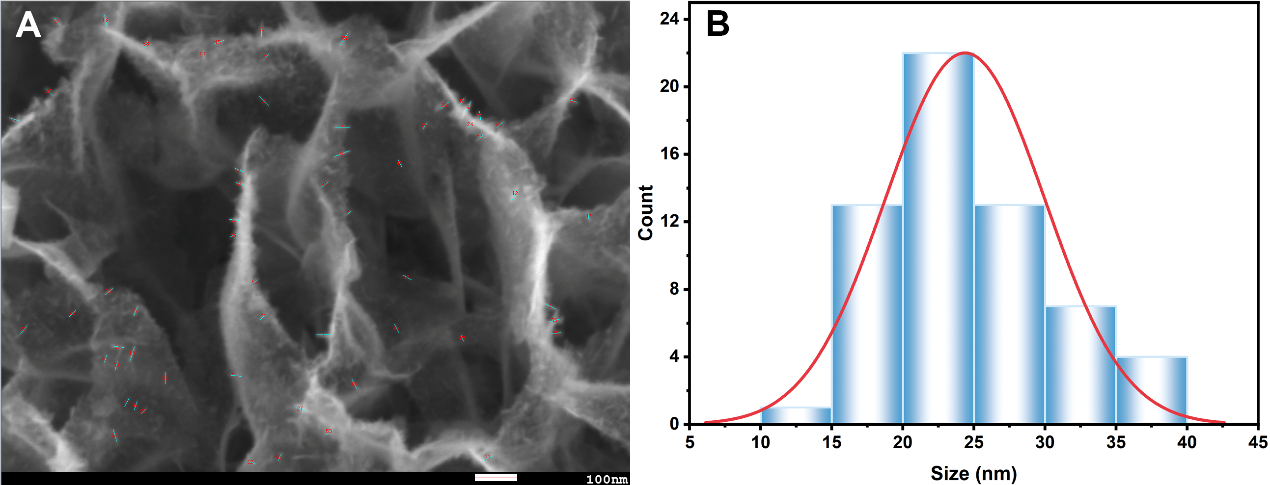
**

**Figure S3.** (A) SEM image of ac-NiCoO NSA with the measured sub-nanostructures being marked; (B) Lateral size distribution diagram of the sub-nanostructures of ac-NiCoO NSA. We measured 60 sub-nanostructures in total and calculated their average value which is 24.4 nm.

**
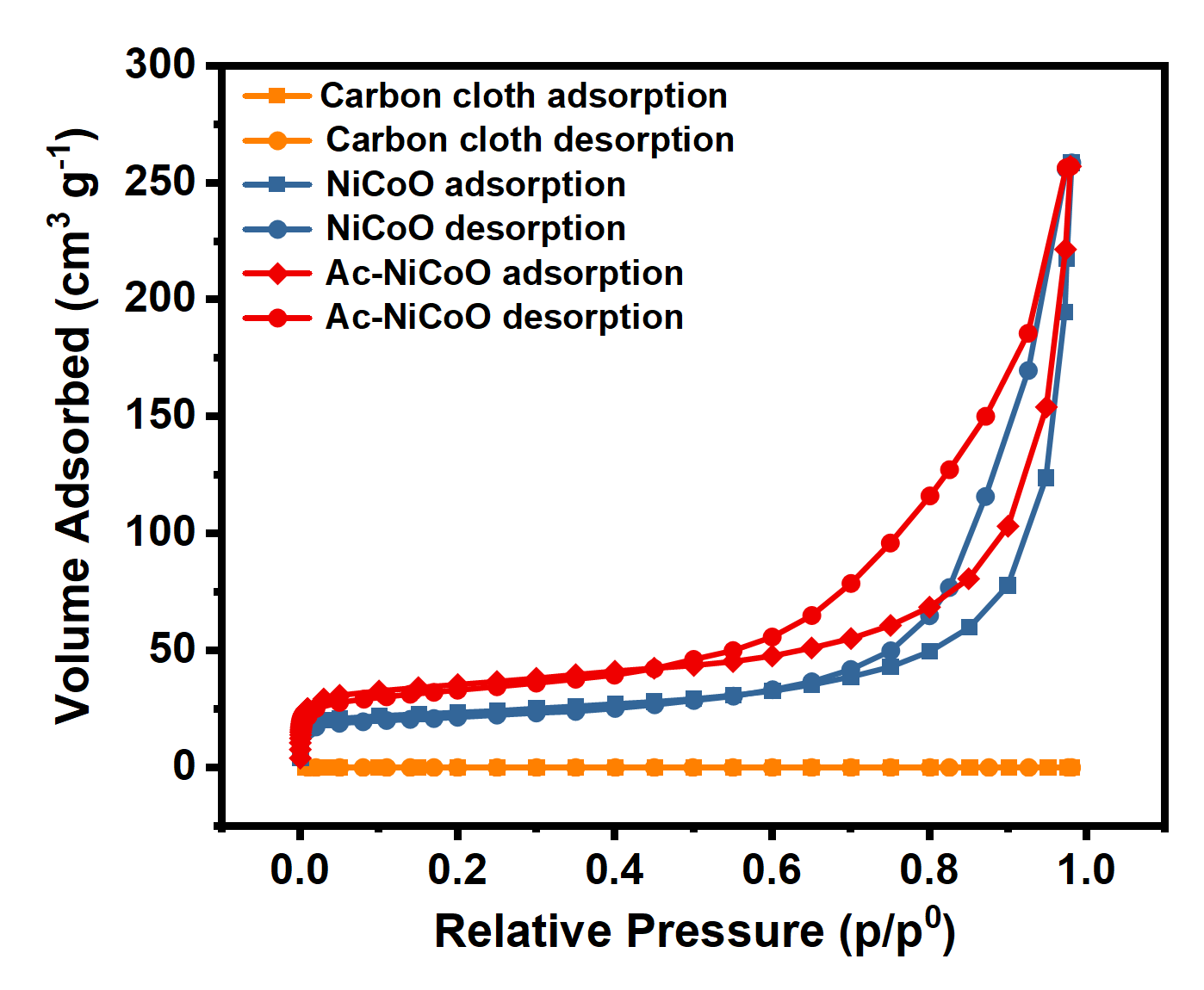
**

**Figure S4.** N_2_ adsorption-desorption isotherm of bare carbon cloth (a), NiCoO (b), and ac-NiCoO (c) at 77 K and pressure of 0 – 1.0 bar, respectively.


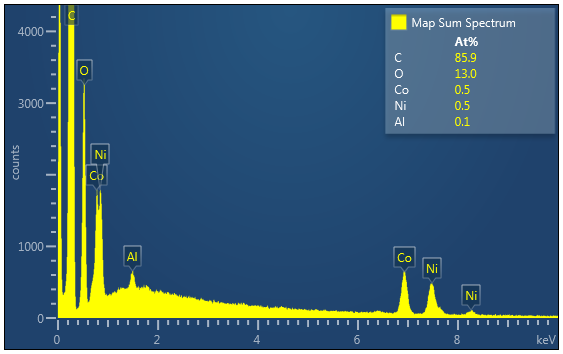


**Figure S5.** EDS spectrum of ac-NiCoO NSA.


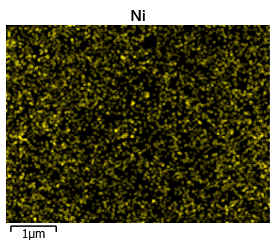

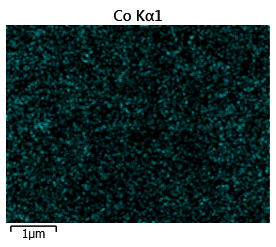

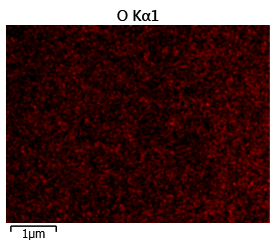


**Figure S6.** Elemental EDS mapping of Ni, Co, and O in ac-NiCoO NSA.


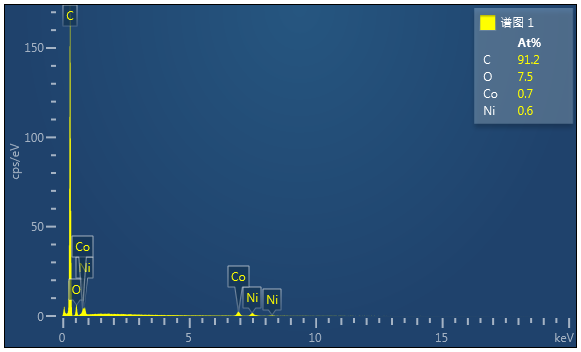


**Figure S7.** EDS spectrum of NiCoO NSA.


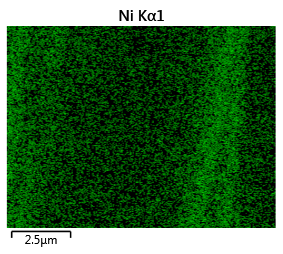

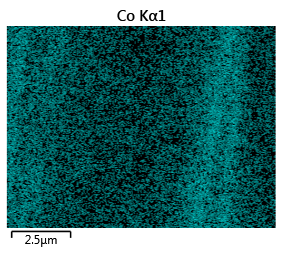

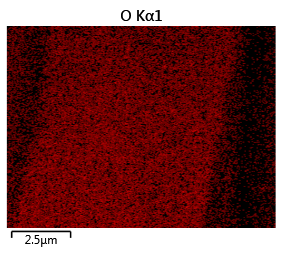


**Figure S8.** Elemental EDS mapping of Ni, Co, and O in NiCoO NSA.

**
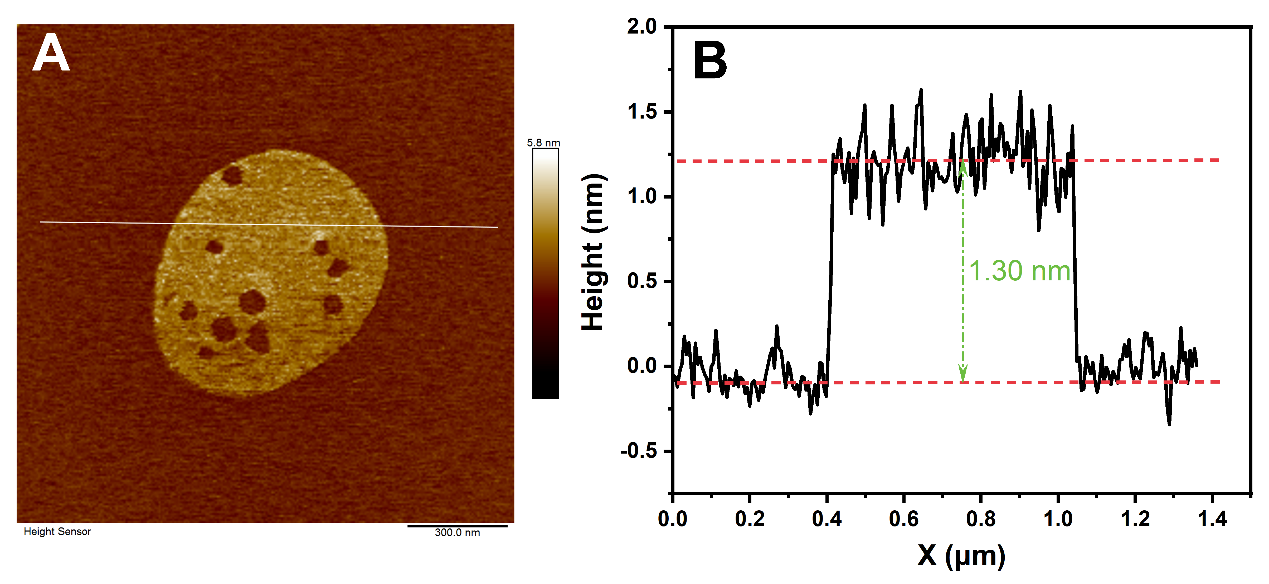
**

**Figure S9.** (A) AFM image of ac-NiCoO NSA and (B) the corresponding thickness disperse curve.

**
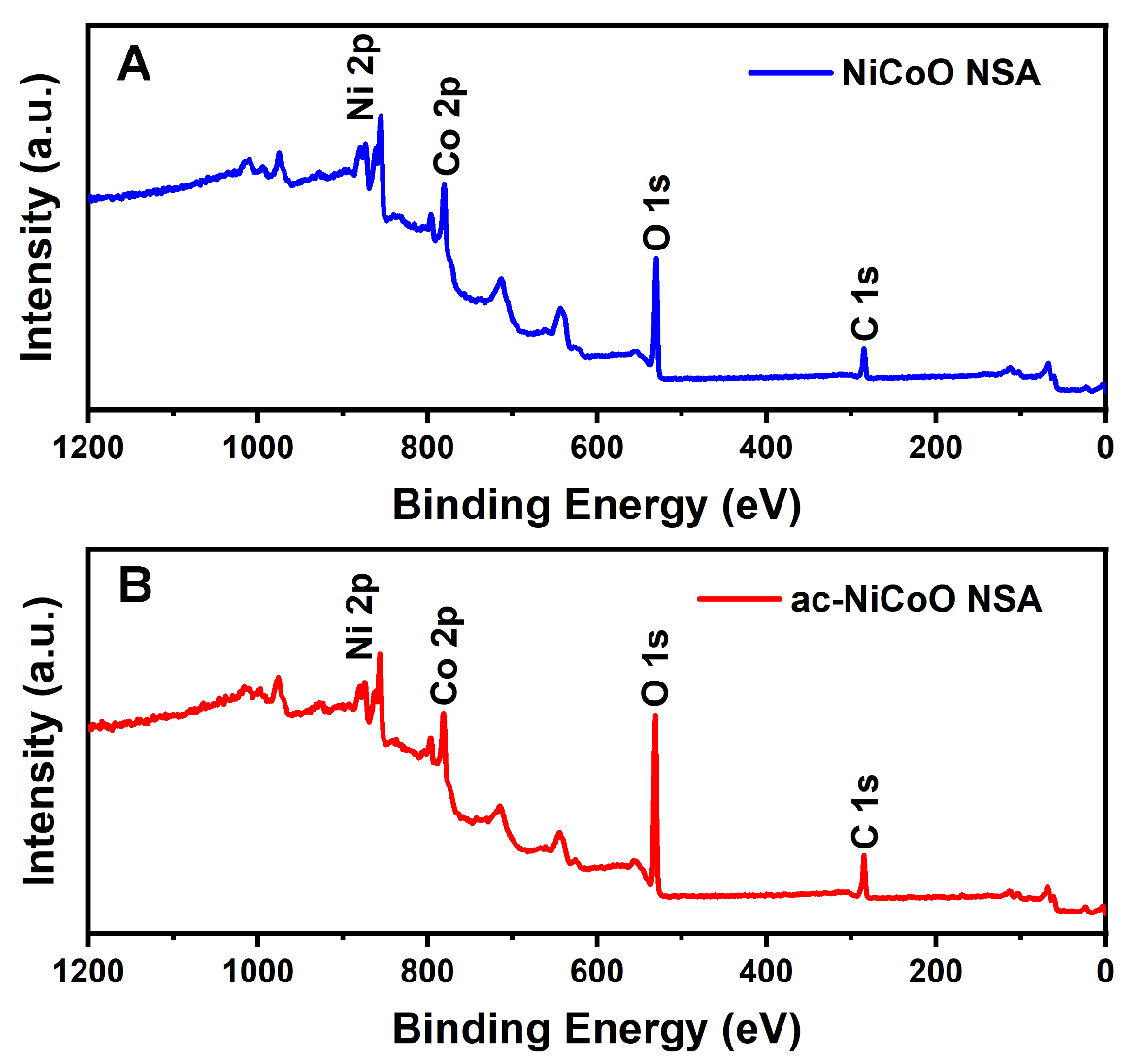
**

**Figure S10.** XPS survey spectra of NiCoO NSA (A) and ac-NiCoO NSA (B).





**Figure S11.** CV curves (20 cycles) of NiCoO NSA at 10 mV s^-1^ during the ECA process with the voltage window of -0.1 – 0.65 V vs Hg/HgO.


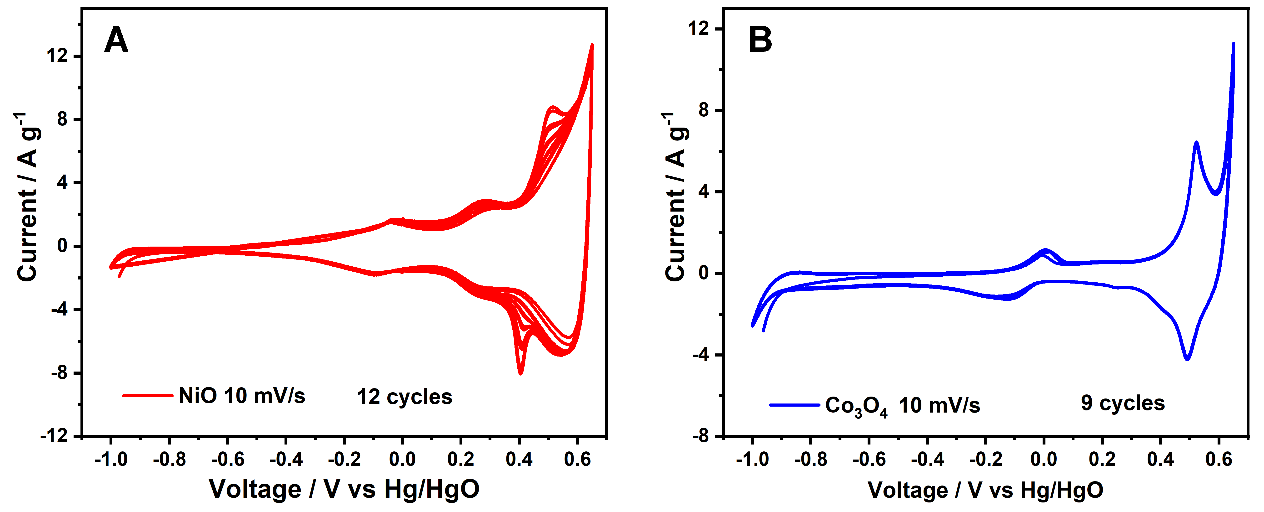


**Figure S12.** CV curves of NiO (A) and Co_3_O_4_ (B) NSAs on CFC substrate under the scanning rate of 10 mV s^-1^.


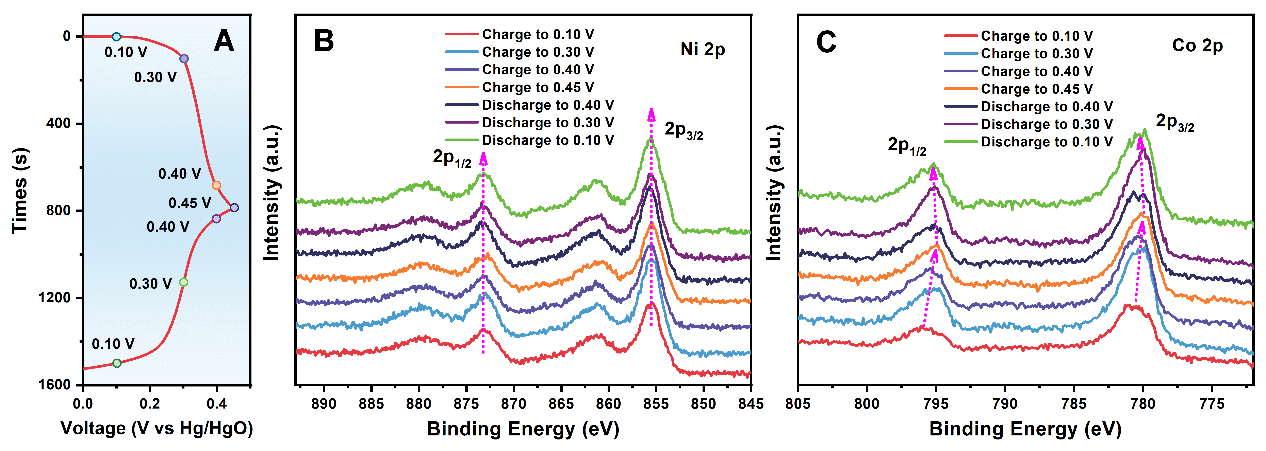


**Figure S13.** (A) GCD curve of ac-NiCoO NSA showing the charge states for XPS measurement; (B and C) XPS spectra of Ni 2p (B) and Co 2p (C) in ac-NiCoO NSA at different charge/discharge states.





**Figure S14.** (a) GCD and CV (b) curves of pristine NiCoO NSA grown on CFC substrate.


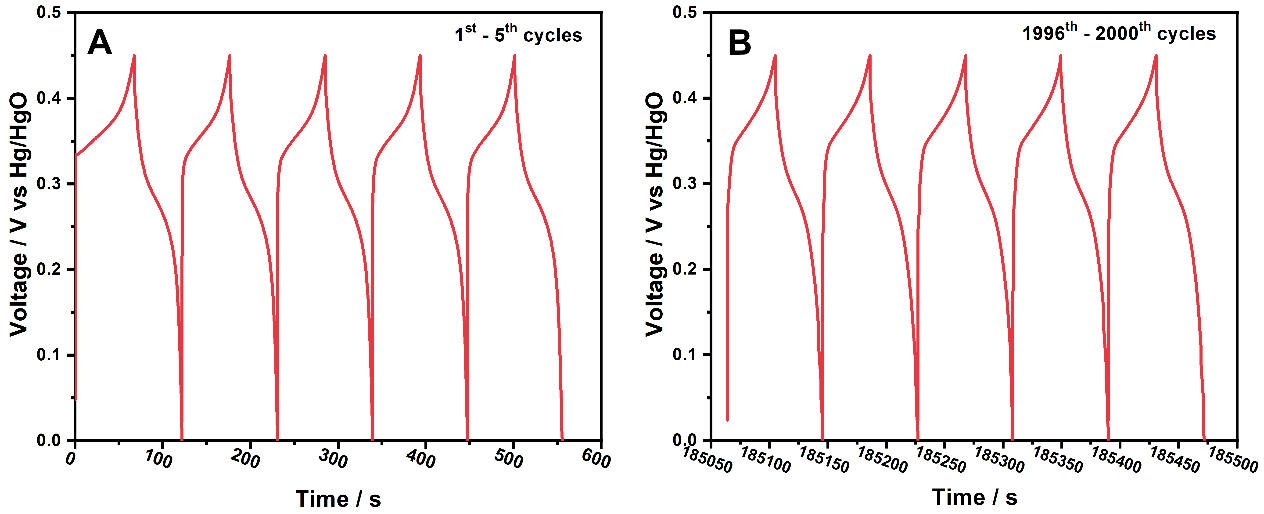


**Figure S15.** The GCD curves showing the 1-5^th^ (A) and 1996-2000^th^ (B) cycles of the ac-NiCoO NSA under the current density of 10 A g^-1^.


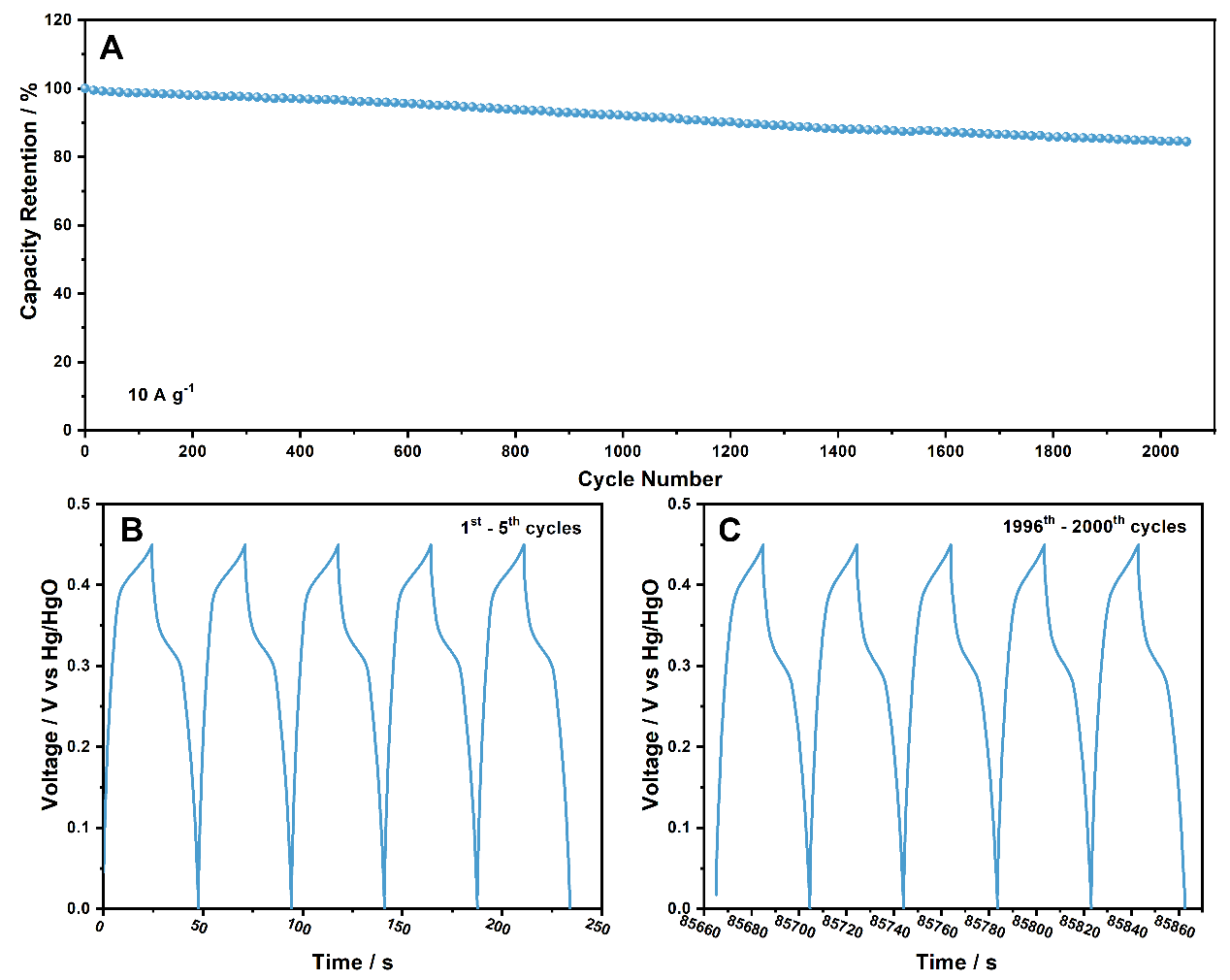


**Figure S16.** The cycling results (A) and corresponding GCD curves showing the 1-5^th^ (B) and 1996-2000^th^ (C) cycles of NiCoO NSA in the voltage window of 0.45 V under the current density of 10 A g^-1^.


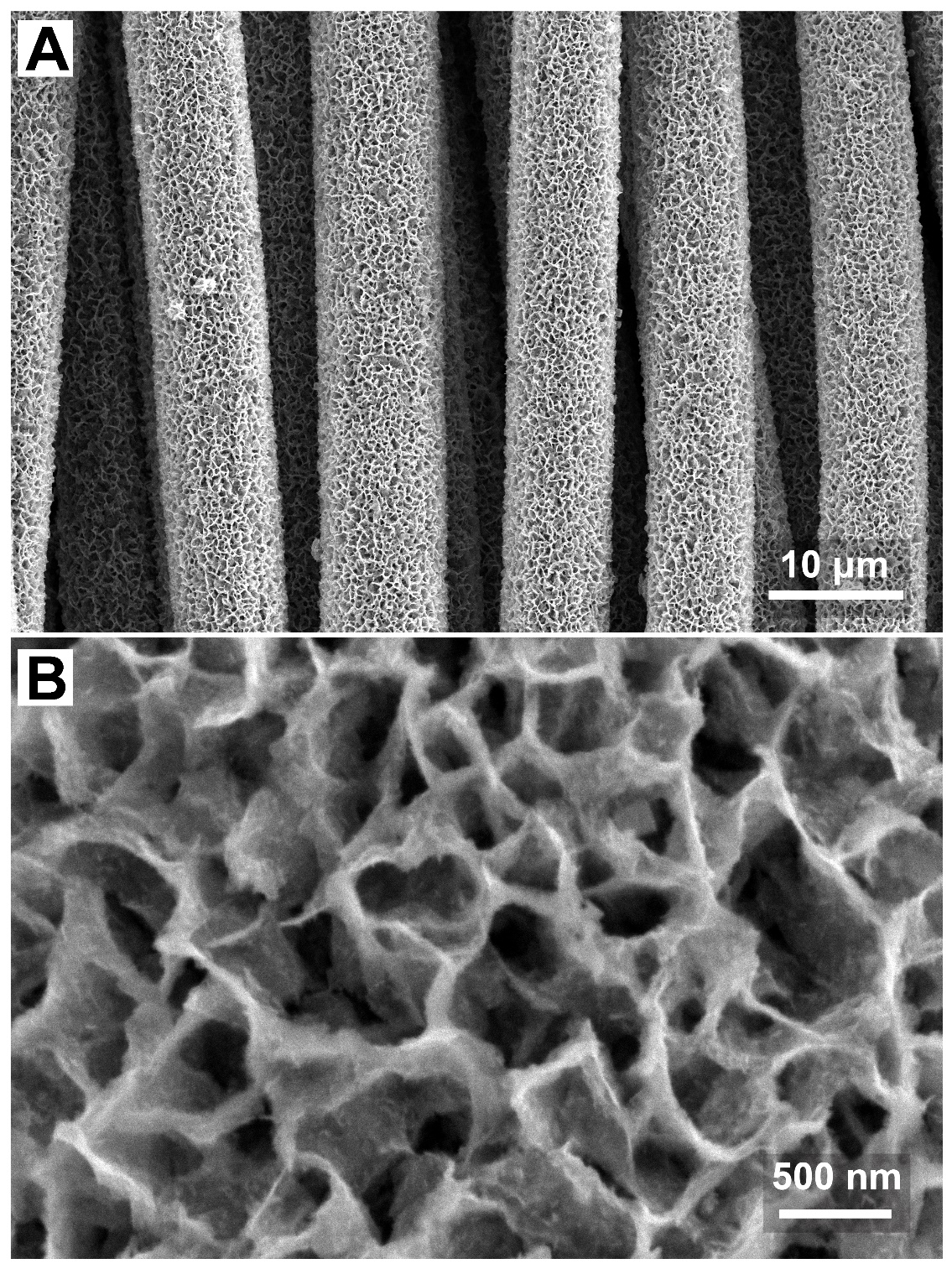


**Figure S17.** The FESEM images of ac-NiCoO NSA after GCD cycling stability test.





**Figure S18.** PXRD pattern of ac-NiCoO NSA material after the cycling test.


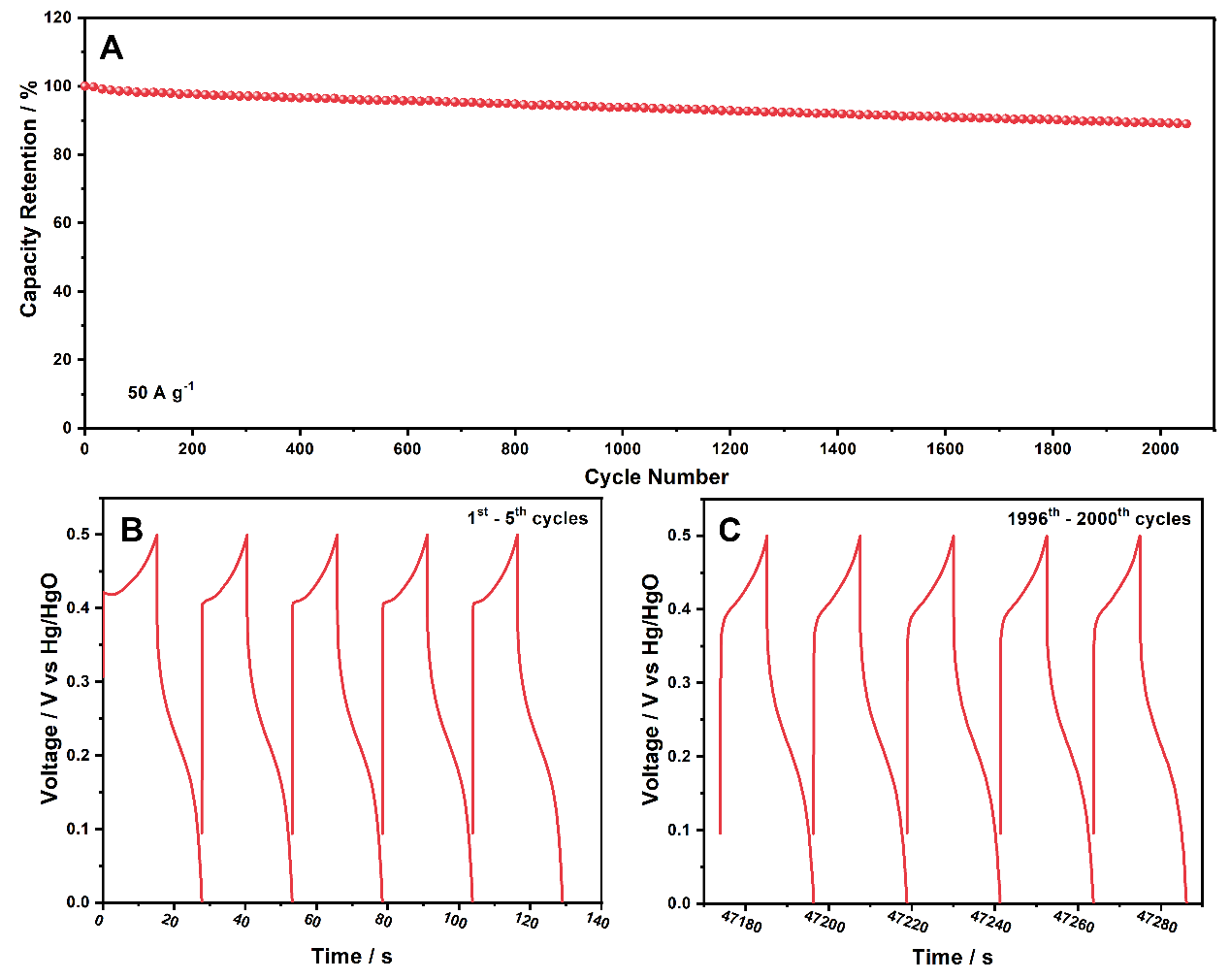


**Figure S19.** The cycling results (A) and corresponding GCD curves showing the 1-5^th^ (B) and 1996-2000^th^ (C) cycles of ac-NiCoO NSA in the voltage window of 0.5 V at the discharge current density of 50 A g^-1^.

**Table S1.** The supercapacitive performance comparison of the state-of-the-art supercapacitors based on transition-metal compounds.

| **Supercapacitors** | **Voltage window (V)** | **Specific capacitance**  **(F g^-1^)** | **Energy density (*E*, Wh kg^-1^)** | **Power density**  **(*P*, kW kg^-1^)** | **Cycling Stability**  **(cycles)** | **Ref.** |
| --- | --- | --- | --- | --- | --- | --- |
| ZnNiCoO//AC | 1.5 | 113.90  (1 A g^-1^) | 35.60  (*P*: 0.19) | 0.94  (*E*: 19.10) | 3000  (94%, 3 A g^-1^) | ^[3]^ |
| NiCo_2_O_4_−NiO//AC | 1.75 | 82.10  (1 A g^-1^) | 34.90  (*P*: 0.88) | 8.75  (*E*: 19.90) | 10000  (86.4%, 2 A g^-1^) | ^[4]^ |
| Mn-NiO NSAs//graphene-CNT | 1.5 | -- | 23.30  (*P*: 0.15) | 4.50  (*E*: 8.80) | 50000  (109%, 6 A g^-1^) | ^[5]^ |
| NiO-CuO//porous graphene | 1.6 | 95.10  (0.5 A g^-1^) | 33.80  (*P*: 0.40) | 8.00  (*E*: 18.40) | 5000  (90.4%, 5 A g^-1^) | ^[6]^ |
| MCP-2 (MgCo_2_O_4_@PPy/NF)//AC | 1.6 | 95.10  (0.5 A g^-1^) | 33.42  (*P*: 0.32) | -- | 10000  (91%, 0.4 A g^-1^) | ^[7]^ |
| NiCo_2_O_4_@MnO_2_//AC | 1.5 | 120.90  (0.25 A g^-1^) | 37.80  (*P*: 0.19) | 7.50  (*E*: 13.3) | 3000  (98.4%, 8 A g^-1^) | ^[8]^ |
| NiCo_2_O_4_@rGO//Ti_3_C_2_T_x_ | 1.5-1.6 | 141.90  (5 mA cm^-2^) | 44.36  (*P*: 0.99) | 12.1  (*E*: 23.53) | 4500  (90.48%, 50 mA cm^-2^ | ^[9]^ |
| FeCo_2_O_4_//GO | 1.6 | 46.40  (0.5 A g^-1^) | 14.5 | 2.18 | 1000  (60%, 1 A g^-1^) | ^[10]^ |
| Mn_2_O_3_/Co_3_O_4_//  NDGH ASC | 1.6 | 92.30  (1 A g^-1^) | 32.80  (*P*: 1.19) | 10.14  (*E*: 18.70) | 7000  (143.9%, 100 mV s^-1^) | ^[11]^ |
| NiCoO-PVA//NiCoO-PVA  (PVA: polyvinyl alcohol) | 1.8 | 42.00  (5 mV s^-1^) | 18.9 | -- | -- | ^[12]^ |
| Mn_0.16_Fe_2.84_O_4_ //AC | 1.5 | 134.00  (1 A g^-1^) | 42.00  (*P*: 0.75) | 20.92  (*E*: 29.00) | 6000  (86%, 10 A g^-1^) | ^[13]^ |
| ac-NiCoO NSA//AC | 1.7 | 113.1 F g^-1^  (0.5 A g^-1^) | 45.40  (*P*: 0.44) | 17.30  (*E*: 20.90) | 20000  (77.4%, 20 A g^-1^) | **This work** |

**References**

[1] D. Zhao, H. Liu, X. Wu, *Nano Energy* **2019**, 57, 363.

[2] L. Q. Mai, A. Minhas-Khan, X. Tian, K. M. Hercule, Y. L. Zhao, X. Lin, X. Xu, *Nat. Commun.* **2013**, 4, 2923.

[3] C. Wu, J. Cai, Q. Zhang, X. Zhou, Y. Zhu, P. K. Shen, K. Zhang, *ACS Appl. Mater. Interfaces* **2015**, 7, 26512.

[4] J. Zhang, F. Liu, J. P. Cheng, X. B. Zhang, *ACS Appl. Mater. Interfaces* **2015**, 7, 17630.

[5] X. Han, B. Wang, C. Yang, G. Meng, R. Zhao, Q. Hu, O. Triana, M. Iqbal, Y. Li, A. Han, J. Liu, *ACS Appl. Energy Mater.* **2019**, 2, 2072.

[6] Z. Fang, S. u. Rehman, M. Sun, Y. Yuan, S. Jin, H. Bi, *J. Mater. Chem. A* **2018**, 6, 21131.

[7] H. Gao, X. Wang, G. Wang, C. Hao, S. Zhou, C. Huang, *Nanoscale* **2018**, 10, 10190.

[8] Y. Zhang, B. Wang, F. Liu, J. Cheng, X.-w. Zhang, L. Zhang, *Nano Energy* **2016**, 27, 627.

[9] A. M. Patil, N. Kitiphatpiboon, X. An, X. Hao, S. Li, X. Hao, A. Abudula, G. Guan, *ACS Appl. Mater. Interfaces* **2020**, 12, 52749.

[10] F. I. Saaid, A. Arsyad, N. S. H. Azman, A. Kumar, C.-C. Yang, T.-Y. Tseng, T. Winie, *J. Electroceram.* **2020**, 44, 183.

[11] H. Ju, X. D. Liu, C. Y. Tao, F. Yang, X. L. Liu, X. Luo, L. Zhang, *J. Alloys Compd.* **2021**, 856, 157134.

[12] P. Siwatch, K. Sharma, N. Manyani, J. Kang, S. K. Tripathi, *J. Alloys Compd.* **2021**, 872, 159409.

[13] X. Tan, R. Wang, X. Liu, W. Wang, L. Cao, B. Dong, *Chem. Eur. J.* **2021**, 27, 9398.
